# Study protocol: the effectiveness and cost effectiveness of a brief behavioural intervention to promote regular self-weighing to prevent weight regain after weight loss: randomised controlled trial (The LIMIT Study)

**DOI:** 10.1186/s12889-015-1869-0

**Published:** 2015-06-04

**Authors:** Claire D Madigan, Kate Jolly, Andrea Roalfe, Amanda L Lewis, Laura Webber, Paul Aveyard, Amanda J Daley

**Affiliations:** Primary Care Clinical Sciences, University of Birmingham, B15 2TT Birmingham, Edgbaston UK; Public Health, Epidemiology and Biostatistics, University of Birmingham, B15 2TT Birmingham, Edgbaston UK; Bristol Randomised Trials Collaboration, School of Social and Community Medicine, University of Bristol, Canynge Hall, 39 Whatley Road, BS8 2PS Bristol, UK; UK Health Forum, Fleetbank House, 2-6 Salisbury Square, EC4Y 8JX London, UK; Nuffield Department of Primary Care Health Sciences, University of Oxford, New Radcliffe House, Radcliffe Observatory Quarter, Woodstock Road, OX2 6GG Oxford, UK

**Keywords:** Weight loss maintenance, Obesity, Public health, Self-weighing, Behavioural medicine

## Abstract

**Background:**

Although obesity causes many adverse health consequences, modest weight loss reduces the incidence. There are effective interventions that help people to lose weight but weight regain is common and long term maintenance remains a critical challenge. As a high proportion of the population of most high and middle income countries are overweight, there are many people who would benefit from weight loss and its maintenance. Therefore, we need to find effective low cost scalable interventions to help people achieve this. One such intervention that has shown promise is regular self-weighing, to check progress against a target, however there is no trial that has tested this using a randomised controlled design (RCT). The aim of this RCT is to evaluate the effectiveness and cost effectiveness of a brief behavioural intervention delivered by non-specialist staff to promote regular self-weighing to prevent weight regain after intentional weight loss.

**Methods:**

A randomised trial of 560 adults who have lost ≥5 % of their initial body weight through a 12 week weight loss programme. The comparator group receive a weight maintenance leaflet, a diagram representing healthy diet composition, and a list of websites for weight control. The intervention group receive the same plus minimally trained telephonists will ask participants to set a weight target and encourage them to weigh themselves daily, and provide support materials such as a weight record card. The primary outcome is the difference between groups in weight change from baseline to 12 months.

**Discussion:**

If effective, this study will provide public health agencies with a simple, low cost maintenance intervention that could be implemented immediately.

**Trial registration:**

ISRCTN52341938 Date Registered: 31/03/2014

## Background

The global prevalence of obesity is estimated to be approximately 24 % [1] and is a significant cause of morbidity in terms of increased risk of type 2 diabetes, cardiovascular disease and many cancers [2–4]. Although many behavioural weight loss treatments are effective in the short term, long term maintenance remains a critical challenge. The period after initial weight loss is when people are at highest risk of weight regain [5]. Few people (1 in 10) recover from even minor lapses of 1–2 kg of regain in weight [5]. Therefore preventing small regains from turning into larger relapses appears critical to achieving effective long term weight control.

### Weight loss maintenance

Compared with weight loss trials, relatively few studies have focused on weight maintenance and those trials that do exist have tended to evaluate intensive interventions. A recent review of RCTs of weight loss maintenance interventions that enrolled obese adults following clinically significant weight loss identified 34 relevant studies [6]. Overall behavioural interventions appeared to be effective, however, it appeared that intervention content, not intensity, was related to effectiveness. There were four important features of effective weight maintenance interventions, all of which are consistent with self-regulation theory [7]; these being goal setting, self-monitoring of weight and behaviour, action plans for weight control through dietary and physical activity behaviours, and plans to deal with risk factors for weight regain and relapse prevention. The review authors also noted that thus far most weight maintenance interventions have been resource intensive and consequently not likely to be scalable. Additionally, information on the cost effectiveness of interventions was very sparse.

### Self-weighing

Given that weight regain after successful weight loss is the norm, we need cost effective weight maintenance interventions. One promising behavioural strategy is regular self-weighing to check progress against a target, a form of self-monitoring. The potential efficacy of self-weighing has been based on the principles of self-regulation theory [7] and the relapse prevention model [8]. There are three distinct stages; self-monitoring, self-evaluation and self-reinforcement [7]. Self-monitoring means systematic self-observation, periodic measurement and recording of target behaviours to increase self-awareness. The awareness promotes and sustains behaviour change through self-evaluation. Self-weighing can provide feedback of how diet and exercise behaviours affect weight and also provide negative or positive self-reinforcement of relevant behaviours. Regular self-weighing may also act as a primer, increasing self-awareness of environmental cues to eat and be inactive [9]. Self-weighing is less cumbersome than monitoring diet and physical activity and may allow people to detect changes in their weight earlier, take immediate action and see the consequences on their weight. It may aid self-management of weight in a manner that could be sustained in the longer term and so have a public health benefit [10].

### Previous research

There have been trials of weight management that have included self-weighing as part a multicomponent programme and these have shown that participants can adhere to daily self-weighing and are effective [5, 11, 12]. However only one trial has included self-weighing as part of a multicomponent intervention to promote weight loss maintenance and the intervention group regained significantly less than the control group at 18 months follow-up [5]. The intervention showed promise, however it would be costly to deliver as it involved face to face sessions and therefore unlikely to be implemented as part of a weight management service within most health systems.

### Pilot work

Previously we have evaluated an intervention to facilitate weight maintenance and prevent weight regain over the longer term in users of a weight management service called Lighten Up [13]. Users of the Lighten Up service were offered a three month weight maintenance intervention after completing their weight loss programme and nine month follow-up data were collected. The intervention focused on encouraging regular self-weighing. Participants who did not own scales were given a voucher to obtain a free set from a local pharmacy and sent a chart to record their weight on a weekly basis and a hints and tips booklet about strategies to facilitate weight management. Participants were telephoned three months later to encourage regular adherence to weekly weighing. The intervention was delivered by call centre staff with no specialist behavioural skills and minimal training. We examined the efficacy of this self-weighing focused weight maintenance intervention on weight regain at nine months by comparing the weight of those offered the intervention (intervention group) (n = 3,290) with participants (n = 478) in the preceding Lighten Up trial [13] who had not received a maintenance intervention (control group). Using intention to treat analysis, both groups regained weight but the intervention group regained 0.7 kg (95 % CI 0.1 to 1.2) less than the control group. In the per protocol analysis, comparing intervention participants who had accepted the maintenance intervention with controls, the mean difference was much larger at 3.0 kg (95 % CI -3.7 to -2.3). Whilst our pilot results were encouraging and offer preliminary evidence to support the intervention, participants were not randomised to the groups, the intervention was not optimally configured to encourage behaviour change, follow-up data were mostly self-reported and the frequency of self-weighing was not recorded.

The effectiveness of the intervention therefore needs to be established with a more robust study. Therefore the primary aim of this study is to evaluate the effectiveness and cost effectiveness of a brief behavioural intervention delivered by non-specialist staff to promote regular self-weighing to prevent weight regain after intentional weight loss. The intervention will be compared with the comparator group. The research will test a theoretically informed weight loss maintenance intervention, following successful completion of standard widely available commercial and National Health Service (NHS) weight loss programmes.

## Methods/Design

### Ethical approval

Ethical approval (ERN_13-138) was reviewed and obtained by the Science, Technology, Engineering and Mathematical review committee at the University of Birmingham, UK. The committee reviewed and approved all study materials on the 29^th^ April 2014 and will approve all study protocol amendments.

### Design

This study was designed in response to a funding call from the National Institute of Health Research Public Health Research Programme in England to answer the research question: “What behaviour change interventions are effective and cost effective in reducing the chance of relapse and sustaining healthy behaviours in smoking, alcohol use, diet or physical activity.” The design is an RCT of 560 adults who will have received a publically funded weight loss programme for 12 weeks and lost at least 5 % of their initial body weight.

### Setting and recruitment

**Fig. 1:**
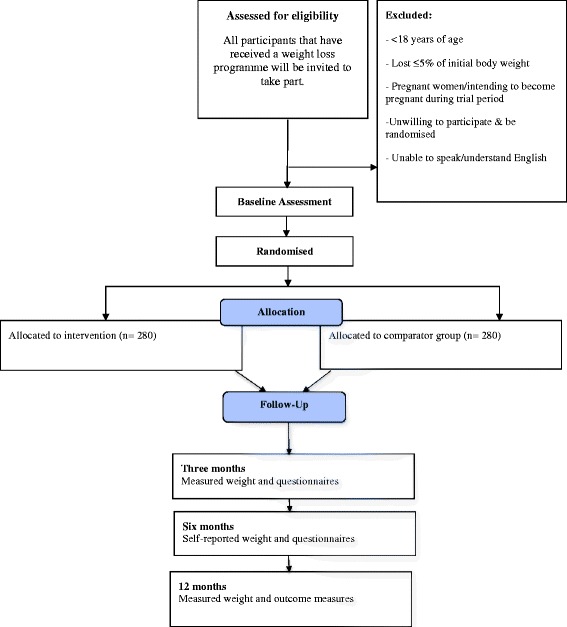
CONSORT flow diagram

All participants in a Public Health funded weight management service will be sent an invitation letter from their service on behalf of the research team, as well as an information leaflet, when they reach week nine of their weight loss programme. The letter will inform participants about this weight loss maintenance study (called LIMIT) and will notify them that as part of the routine service they will be asked whether they are willing to take part in a study to prevent them regaining the weight they may have recently lost. At week 11 of the weight loss programme participants will be asked to report their current weight to enable the research team to calculate the total amount of weight loss since starting their weight loss programme. Those who have lost at least 5 % of their starting weight will be asked by members of the research team to participate in a study about preventing weight regain. Figure 1 depicts participant flow through the trial.

### Inclusion criteria

- Aged 18 years or more.
- Own a mobile phone or landline phone that can receive SMS text messages.
- Able to understand English sufficiently to complete the study procedures.
- People who have lost at least 5 % of their starting weight at the end of their weight loss programme. The initial screen for 5 % weight loss is based on self-reported weight at the baseline screening call and is later verified objectively at the baseline home visit. Because we anticipate there is likely to be variation in participants self-reported weight using their home scales and the calibrated scales used by researchers at the home visit to verify percentage weight loss, the decision was made to allow some flexibility in the inclusion criteria at the home visit and include people who were confirmed to have lost at least 4 %.

### Exclusion criteria

Women who are known to be pregnant or intending to become pregnant during the study.

### Comparator group

The comparator group receive the standard Lighten Up maintenance leaflet which consists of a list of tips that may help people to maintain their weight under the main headings of: “Managing your thoughts and any slip ups, planning, shopping, eating behaviour, support, coping with social occasions and rewards”. Participants will also receive a diagram representing healthy diet composition and a list of websites related to weight management.

### Intervention group

The intervention group receive support telephone calls at weeks zero, two and four that encourage daily self-weighing, recording of weight on a record card, setting a weight target together with regular reminder SMS text messages. The telephone calls aim to encourage and provide minimal behavioural intervention to support participants to weigh themselves daily and record their weight on the weight record card, which are given to participants at the initial baseline visit.

#### Intervention components

The goal of the intervention will be for participants to make a commitment to avoid regaining more than 1 kg of their baseline weight. The main element used to achieve both goal setting and the monitoring necessary to ensure this, is support telephone calls at weeks zero, two and four, that encourage target setting and daily self-weighing, together with reminder text messages three days per week for the first four weeks, reducing to twice weekly thereafter. We have purposefully designed the intervention such that the telephone contacts and frequent texts messages occur in the first four weeks because the period after initial weight loss is when people are at highest risk of weight regain [5]. We also wanted to maximise the possibility that regular self-weighing becomes a habit for participants. Table 1 details the behaviour change techniques used based on the CALO-RE Behaviour Change Taxonomy [14].

**Table 1:** Intervention components using the CALO-RE behavioural change taxonomy [14]

Behavioural technique	Definition
*Goal setting (outcome)*	Telephonists encourage participants to set a weight goal for regain such as ‘*In a year I aim to weigh no more than I do now’.*
*Prompt review of outcome goals*	Participants will be instructed to remain within 1 kg of their study baseline weight and to review their weight each day against this target.
*Provide information on the consequences of behaviour in general*	Telephonists discuss the benefits of self-weighing with the participant.
*Environmental restructuring*	The telephonist encourages the participant to cue this behaviour ‘*move the scales into your bathroom so when you see them after your shower it will remind you*’.
*Provide information on where and when to perform the behaviour*	The telephonist asks participants to describe when and where the weighing will take place. Participants will be encouraged to weigh themselves at the same time every day.
*Use follow-up prompts*	Participants receive telephone calls at weeks zero, 2 and 4 that encourage daily self-weighing, together with reminder text messages every other day for the first four weeks, reducing to twice weekly thereafter.
*Barrier identification/ Problem solving*	The telephonists offer practical solutions and give participants ideas and strategies to overcome barriers to daily self-weighing. Participants will be advised that if their current weight is more than 1 kg above target weight then they would be best to restart following the plan they followed for eating and physical activity when they were on their weight loss programme.
*Agree behavioural contract*	The telephonist asks participants if they can commit to a weight change target and to daily weighing.
*Provided general encouragement*	The telephonist encourages the participant ‘*remember every time you record your weight you are one step nearer to this becoming a healthy habit’*’
*Prompt self-monitoring of behavioural outcome*	Participants will be advised to weigh themselves daily and record it on the record card provided
*Prompt social support*	Prompt participants to ask someone they care about to support them. Participant are advised to tell this person their goal and ask them to remind the participant of this goal and check commitment to it and whether it has been achieved every week.

#### Telephone calls

The telephonists will call participants three times; at weeks zero, two and four of the intervention. The calls will be about five minutes each. The aim of the intervention is to prevent participants regaining more than 1 kg, however we recognise that some participants will want to continue to lose weight and we will advise participants to set a weight loss goal for 12 weeks. If participants wish to continue to lose weight we will advise these participants to aim to lose 1–2lbs per week as in line with national guidance [15]. Participants will be encouraged to weigh themselves at the same time every day wearing similar amounts of clothing or no clothing. The telephonist explains that the aim of weighing frequently is to check themselves against the target weight set. The telephonist will ask participants to write their target weight on their record card provided and explain that every day the participant should check their recorded weight against the target weight. The telephonist will encourage the participant and advise them that if their current weight is more than 1 kg above their weight at the end of their weight loss programme then they should restart following the plan they followed for eating and physical activity when they were on their weight loss programme.

#### Week two and four calls

The telephone calls in weeks two and four will include a review of how participants are getting on compared to their target weight, the frequency of self-weighing and the recording of weight over the previous weeks. Those not weighing themselves daily will be asked about barriers to this and the telephonist will help with practical solutions/ideas and strategies of how they might overcome them. Participants will be further encouraged to self-weigh daily. The importance of using the weight record to compare their weight to their goal will be emphasised, as well as engaging in regular physical activity/healthy eating. The aim is to suggest to participants that daily weighing is a healthy habit to help them manage their weight and that they should adopt this for the rest of their life. Apart from these scheduled calls, participants are not able to telephone the call centre to receive further support.

### SMS text messages

Automated reminder SMS text messages will be sent three times per week for the first four weeks, reducing to twice weekly for eight more weeks. The goal for the intervention is to encourage self-weighing such that it becomes a habit to prevent weight regain. We will send reminder texts twice per month as a ‘top up’ strategy after the 12 week intervention until 12 months follow-up. Participants cannot reply to the texts and they provide no tailored or interactive component.

#### Delivery of the intervention

Gateway Family Services are a triage centre for weight management services on behalf of Birmingham Public Health Directorate, England. The Gateway call centre is staffed by employees who are trained in call centre management systems and customer relations, but not in nutrition or weight management. Call centre staff do not offer any opinions or undertake any motivational interviewing, but they listen, offer positive reinforcement about regular self-weighing and setting weight goals, offer advice about intention implementations, give encouragement and pass on factual information. Call centre staff will be given a detailed intervention manual from the University research team and will be given up to five practice calls to enable simulation of intervention calls and for the research team to provide feedback about different methods to communicate to people about their weight. The University research team will also be available if any queries arise throughout the trial.

### Primary outcome

The difference between the groups in weight change from baseline to 12 months follow-up.

### Secondary outcomes

1. The proportion of participants in the intervention and comparator group who have regained less than 1 kg in weight at three and 12 month follow-up.
2. The difference between groups in weight change from baseline to three months follow-up (i.e., end of behavioural support for weight maintenance).
3. The cost to the NHS per kg, and per kg/m [2], of the additional weight loss maintained for the intervention compared to comparator group at 12 months, the cost per quality adjusted life years (QALY) during the intervention period and predicted lifetime QALYs gained.
4. To assess the effects of the intervention on uncontrolled eating, emotional eating and weight preoccupation at three and 12 months follow-up.

### Non-efficacy outcomes

1. The difference in frequency of self-weighing in the comparator and intervention groups at three and 12 months.
2. Using items from the three factor eating questionnaire we will examine if there are differences in cognitive restraint of eating and frequency of self-weighing at three, six and 12 months [16].
3. Dose–response of frequency of self weighing on weight change at three and 12 months.
4. The association between automaticity (measured by HABITS index) and frequency of weighing and the association of weight change and automaticity at three and six months follow-up.

### Assessments

#### Baseline

A researcher measures participant’s height and weight at a home visit before randomisation. Participants complete the baseline questionnaire and answer the open ended questions about their previous weight loss attempts and strategies they use to control their weight before being randomised. As a process measure the intervention group will be given a set of BodyTrace scales to use to weigh themselves, these scales transmit weight data to a remote database to provide an objective measure of weighing frequency (see below).

#### Three and 12 month follow-up

Participants will be mailed the follow-up questionnaire prior to being contacted to arrange a visit by the research team. At each visit the questionnaire is collected and participants will be weighed by the researchers. Participants will be given a £20 high street shopping voucher for completing each follow-up.

#### Six month follow-up

Participants will be sent the six month questionnaire and a self-addressed envelope to return in the post.

### Process measures

We are collecting process measures to monitor recruitment, intervention delivery and potential variables that may mediate the outcomes. Our hypothesis is that self-weighing leads to the development of conscious cognitive restraint, which the National Weight Control Registry has found to be a key behavioural attribute associated with weight maintenance [17]. Using six items from the revised three factor eating questionnaire [16] we will examine if feedback from self weighing has led to development of conscious cognitive restraint of eating (measured in both groups) at three, six and 12 months post randomisation. In the intervention group we will use seven items from the index of habit strength [18] to measure the automaticity of self-weighing and five items from Steinberg [12] to measure perceptions of daily self weighing at the same follow-ups. At three, six and 12 months follow up participants in both groups will be asked to complete a series of open ended questions about any weight control practices they may have used in the previous two weeks, which will be coded. Within these questions we will specifically ask participants whether they have reapplied any behavioural techniques they acquired through participation in their weight loss programme and whether they have re-enrolled in a weight loss programme. We will then be able to examine whether the intervention may work by encouraging people to reapply lessons and techniques they learnt on the weight loss programme or it works by encouraging participants to enrol in a weight loss programme once more. Table 2 documents the process measures collected.

**Table 2:** Measurements and study questionnaires at baseline and follow-ups

Reach		
Number of participants eligible		
Number of participants consented		
Number of participants consented and randomised		
Intervention		
	Comparator group	Intervention group
Frequency of recording self-weighing –record cards		X^a^
Frequency of self-weighing (objective scales)		X
Psychological Measures		
Index of habit strength [18]		X
Energy restriction [16]	X	X
Perceptions of self-weighing [20]		X
Thoughts about regular weighing (open ended questions)		X
Weight locus of control [21]	X	X
Weight loss strategies		
Weight control strategies (including frequency of self-weighing) [22]	X	X
Attendance at commercial weight loss programmes & application of skills learnt at weight loss programme (open ended)	X	X
Open ended questions asked if participants are no longer managing their weight.	X	X
Intervention delivery of phone calls		X^a^

^a^3 month follow-up only

One weakness of previous trials is that self-weighing frequency was based on self-report rather than objective measures. Here we use Body Trace scales (http://www.bodytrace.com/) which use mobile phone signals to send the weight of the participant to a central database in real time. This will provide an objective measure of the weighing frequency of intervention participants and allow us to examine adherence to the daily weighing intervention.

### Sample size

The standard approach to sample size calculation is to aim to detect a worthwhile intervention effect. This is difficult here because there is a linear relation between overweight and mortality (30 % increase per 5 kg/m [2]) [19]. This kind of intervention may be applied broadly in contexts outside of the specific behavioural intervention we propose here and in any case, even here, at its likely maximal intensity and cost, is likely to cost around £20 per person or less in public health practice. Even very small decrements in weight are likely to be cost effective if they are maintained throughout life. Consequently, instead of an approach where we specify a sample size based on a clinically important difference, we propose a sample size based on the likely size of effect we expect to achieve, a 2 kg difference in change in weight at 12 months. A total of 280 participants randomised to each group (n = 560) will be sufficient to detect a 2 kg difference in change in weight at 12 months follow-up between the intervention and comparator group, with 90 % power and 5 % significance level. This estimate is based on data from our pilot trial [13] where we found the standard deviation of the difference from baseline (i.e., end of weight loss/start of maintenance programmes) to nine months follow-up in those who lost at least 5 % of their starting weight was 6.3 kg. 560 participants allows for 25 % loss to follow-up at 12 months.

### Randomisation

The randomisation list will be developed by an independent statistician within the Primary Care Clinical Research and Trials Unit using NQuery Advisor. Participants will be randomised to the intervention or comparator groups on a 1:1 basis using random permuted blocks of varying size. Some people who have lost weight will want to continue attending the weight management group and pay themselves, after completing the NHS-provided 12 week course, and this is likely to have an important effect on weight at 12 months. To ensure this effect is balanced by treatment arm we will stratify randomisation by whether participants intend to continue with their weight loss programme or not.

Participants will be randomised at the baseline appointment after consent is gained and eligibility checked. The research assistants will be operating in participants’ homes and out of office hours so the only option available will be to implement randomisation by opaque sealed envelopes. These will be sequentially numbered and research assistants open these in sequence and the trial manager checks their adherence to this instruction regularly.

### Blinding

In the information sheet participants will not be told that this is a trial about target setting and daily weighing and will therefore be blinded to group allocation. Instead they will be told that it is a trial of different strategies to prevent weight regain after weight loss. After randomisation the intervention group will know they have been allocated to the weighing group as they will receive a set of weighing scales but the comparator group will remain blinded that the intervention group are being asked to weigh themselves daily. We will also ensure researchers taking follow-up outcome measures will be blinded to group allocation by providing the trial documents in a sealed envelope with a sticker on the front to record the weight of the participant prior to asking questions specific to their trial arm, which inevitably would reveal the random allocation. The researcher undertaking weight measurements (primary outcome) will not have collected baseline or three month measurements therefore remain blind to group allocation. Participants will be asked not to reveal their group allocation to researchers at follow-up. The trial statistician will remain blinded to group allocation until analysis is completed.

### Analysis plan

The analyses will be conducted using the intention to treat principle. The primary outcome will be assessed by analysis of covariance to compare weight change (baseline to 12 months) between the groups. Thus the analysis uses weight at follow-up adjusted for baseline weight and adjusts for the baseline stratification factor, intention to maintain or continue weight loss, as is standard. All participants will be included in the primary analysis but those who are absent from follow-up will have final weight imputed. A similar analysis will be used to compare the secondary outcome of change in weight from baseline to three month follow-up.

The analysis of the proportion of participants in each group who regain no more than 1 kg from their weight at the end of the weight loss programme (i.e., successful maintainers) at three and 12 months follow-up will be conducted using generalised mixed modelling adjusting for the stratification variable. The results will be presented as odds ratios, 95 % confidence intervals and associated p values.

We have four pre-planned exploratory subgroup analyses, examining the effect of gender, initial weight loss programme, whether a person is intending to continue to attend their weight loss programme or not and whether a participant wished to lose or maintain weight. It is possible that different weight loss programmes teach people weight loss maintenance skills to a greater or lesser extent and therefore this could modify the effectiveness of the intervention. Gender may also influence the effectiveness of the intervention as previous research has found men lose more weight than females and have had less attempts at weight loss which could influence subsequent maintenance of weight loss [13, 23]. As some participants will wish to continue to lose weight or continue to attend their weight loss programme the intervention we propose here may not be as effective for those that are aiming to lose weight, we will explore whether there is support for this hypothesis.

The effects of the intervention on uncontrolled eating, emotional eating and weight preoccupation at three and 12 months will be investigated. Groups will be compared with repeated measures mixed modelling, adjusting for stratification variable. The results will be summarised as adjusted means and 95 % confidence intervals.

### Analysis plan for process measures

We are likely to include the following analyses to examine potential process variables. We will examine the degree to which participants develop automaticity by the habit index and the regularity of weighing assessed by their objective weighing scales and their association with weight change. Furthermore, we hypothesise that regular weighing induces participants to develop conscious cognitive restraint over their food intake and we will therefore assess the association between the frequency of weighing and the development of conscious cognitive restraint. As these are repeated measures we will use repeated measures mixed modelling. A Logic model will be developed and full mediation analysis will be undertaken using either Baron and Kenny’s approach or structural equation modelling.

The potential dose–response on the weight change at three and 12 months will be explored by including the frequency of self-weighing (average times per week) and its interaction with intervention arm in the mixed model analysis.

### Serious adverse events requiring hospitalisation

We will summarise the occurrence of serious adverse events in each arm relating to bulimia, anorexia and self-harm due to body dissatisfaction that result in hospitalisation by percentages and 95 % confidence intervals.

### Cost effectiveness modelling

We will use a model to examine the cost-effectiveness of the intervention over a person’s lifetime. The model is the UK Heart Forum’s “Obesity Micro-simulation Model”. At present the model estimates the future burden of diseases by making evidence based extrapolations of selected risk factors specific to the following BMI related diseases; hypertension and stroke, diabetes mellitus type 2, myocardial infarction, osteoarthritis and obesity associated cancers. The micro-simulation incorporates a sophisticated economic module. The model employs Markov-type simulation of long-term health benefits, health care costs and cost-effectiveness of specified interventions. It synthesises and estimates evidence on cost-effectiveness analysis and cost-utility analysis within the countries. The model is used to project the differences in quality adjusted life years (QALYs), lifetime health-care costs and as a consequence of interventions incremental cost effectiveness ratios (ICERs). Sensitivity analysis is also conducted within this model.

## Discussion

Many people would benefit from losing weight but intensive interventions cannot be delivered to this number of people due to the high costs of such interventions. Even if our strategy leads to a smaller effect than a complex intervention, the higher potential reach of the simple cheap intervention we are testing here would still result in substantial gains to public health. If we have a range of simple evidence-based self-help strategies that may prevent weight regain we can encourage the public to use these outside of formal programmes. This study will provide public health agencies with a simple, ‘ready to go’, low cost weight loss maintenance intervention that could be easily implemented, if effective. We purposefully designed this trial to ensure that there is a high ecological validity. We have created a manual for non-specialist staff to implement this intervention and are using this kind of staff in the trial.

### Considerations for future research

These experiences have also raised questions about when is the best time to intervene for weight loss maintenance. From a practical service delivery perspective, participants receive a 12 week weight loss programme commissioned by Public Health and it is hoped that people continue with their weight loss, however unfortunately we know that weight regain occurs. Therefore we need to find effective interventions that are offered at the end of weight loss programmes. The challenge however is that many participants in weight loss programmes will not lose sufficient weight to achieve their prior goals or to attain a healthy weight. Therefore many people will not even consider that maintaining their current weight is a worthwhile goal. This may need to be considered in future intervention development and implementation.

In conclusion, the LIMIT study will test whether a simple low cost intervention that is easily implementable is effective in preventing weight regain in people that have recently lost weight.
